# Supplementary material for: Systematic analysis of drug-associated myocarditis reported in the World Health Organization pharmacovigilance database
Source: Nat Commun. 2022 Jan 10;13:25. doi: 10.1038/s41467-021-27631-8 (PMC8748719; doi:10.1038/s41467-021-27631-8)

Supplementary Material

For “**Systematic analysis of drug-associated myocarditis reported in the WHO pharmacovigilance database**” by LS Nguyen et al.

Supplementary Figure 1. Overlap between drug classes in the dataset. In this figure, we observe 2 reports with a case of drug-associated myocarditis related to both cytotoxic drugs and immunotherapy.

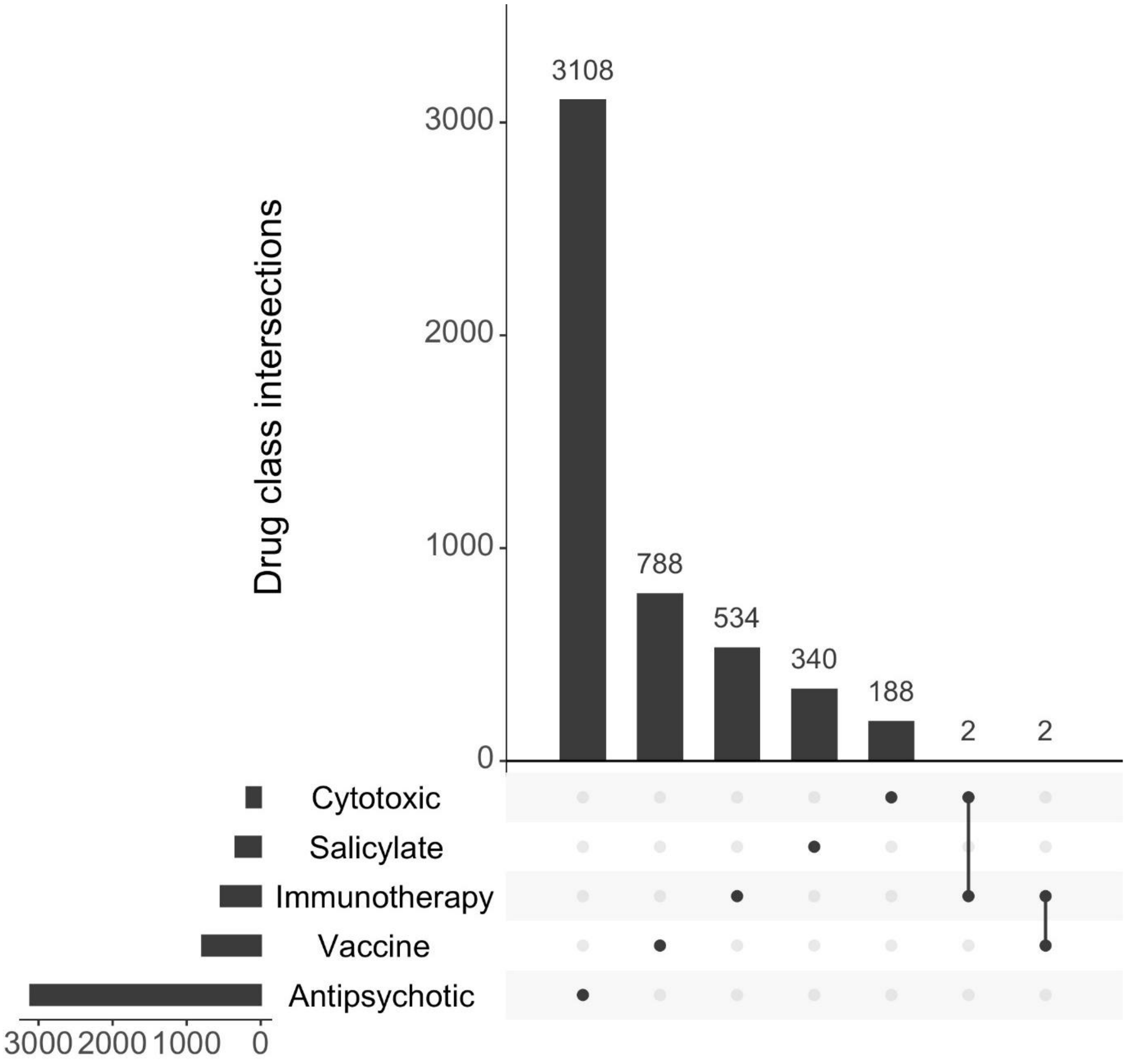

Supplementary Figure 2. Overlap between drug substances in the antipsychotic group

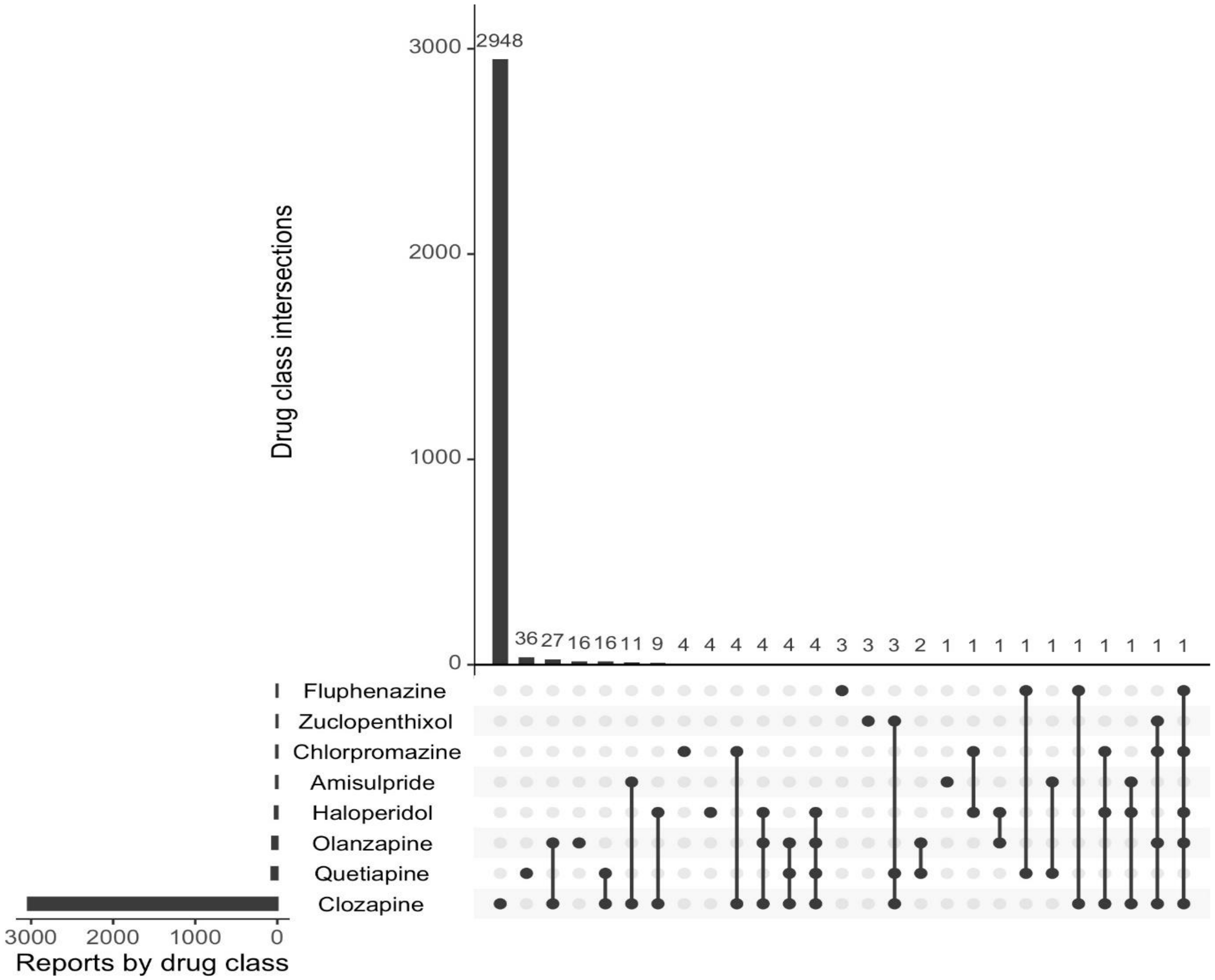

Supplementary Figure 3. Overlap between drug substances in the salicylate group

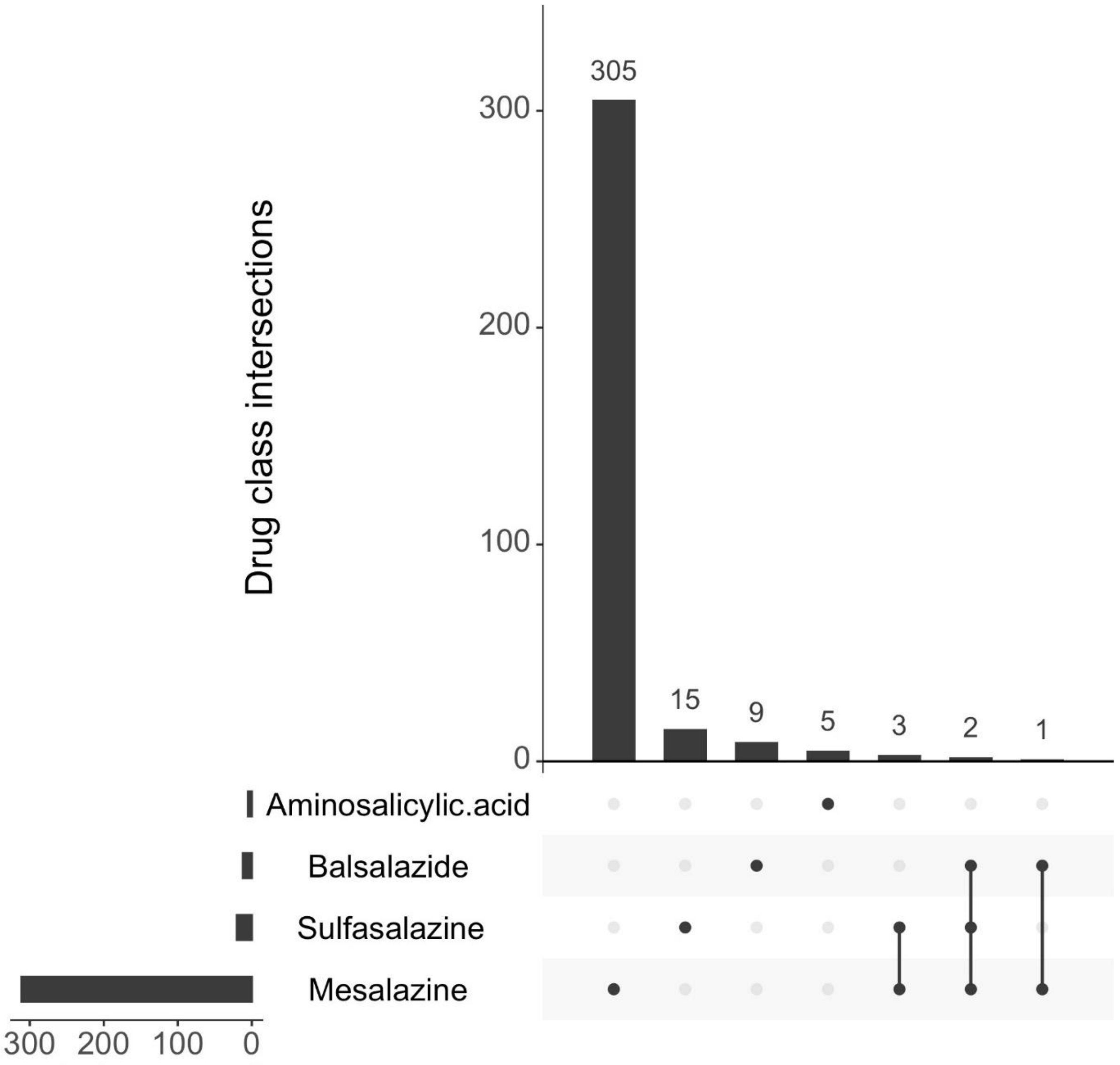

Supplementary Figure 4. Overlap between drug subclasses in immunotherapy group. Subclasses in immunotherapy regroup anti-PD1 (cemiplimab, nivolumab and pembrolizumab), anti-PDL1 (atezolizumab, avelumab and durvalumab), anti-CTLA4 (ipilimumab) and interleukin-2 (aldesleukin).

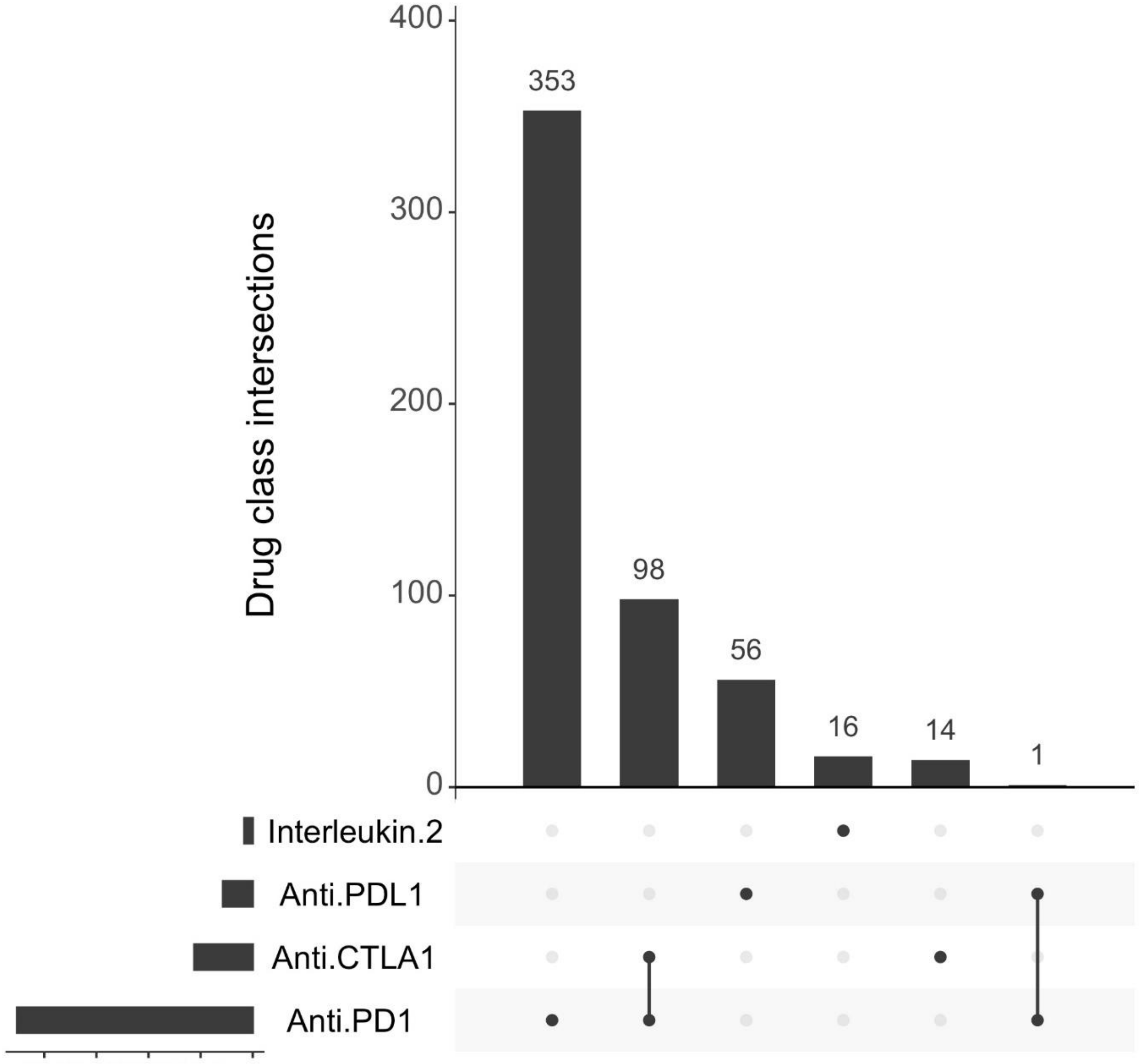

Supplementary Figure 5. Overlap between drug subclasses in cytotoxic group. Subclasses in cytotoxic regroup alkyating agents (busulfan and cyclophosphamide), anthracyclines (daunorubicin, doxorubicin, epirubicin and idarubicin) and antimetabolites (cytarabine and fluorouracil).

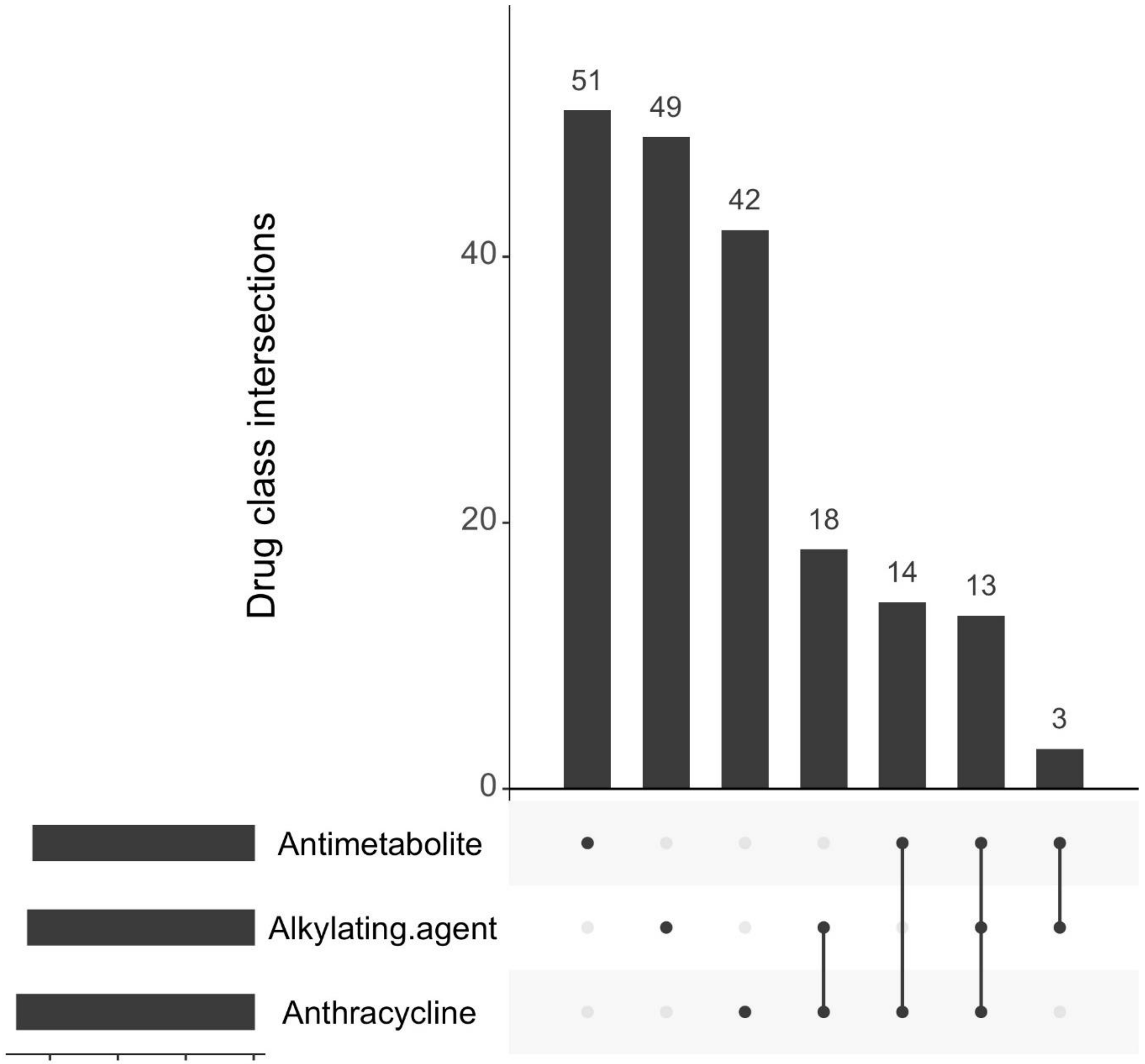

Supplementary Figure 6. Overlap between types of vaccines in the vaccine group (panel F). Abbreviations: DTPP: diphteria, tetanos, pertussis and/or polio vaccine; HepA/HepB: hepatitis A and/or hepatitis B vaccine.

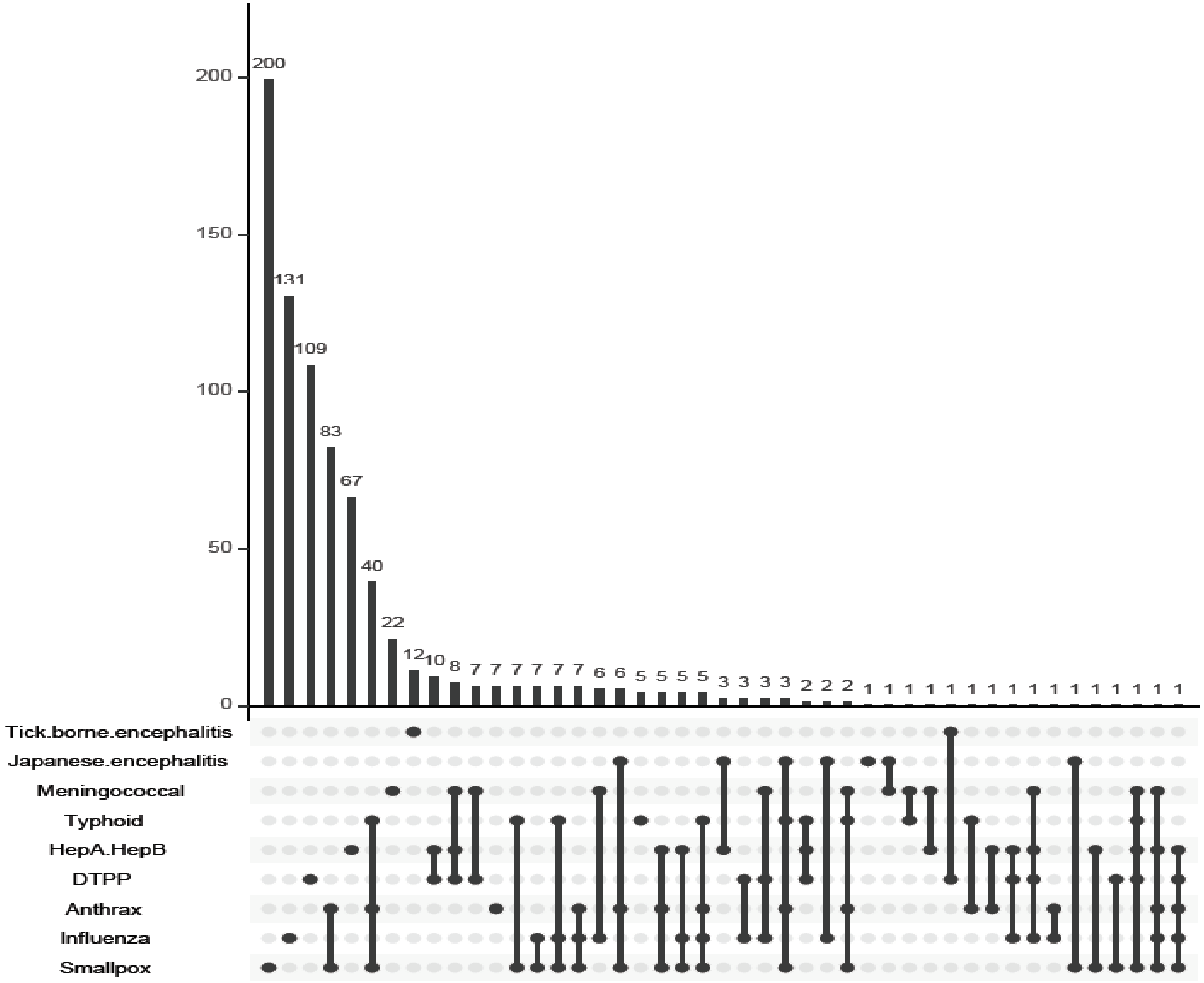

Supplement: Supplementary file 1 — Supplementary Information [file 41467_2021_27631_MOESM1_ESM.pdf]
