# Supplementary material for: Systematic analysis of drug-associated myocarditis reported in the World Health Organization pharmacovigilance database
Source: Nat Commun. 2022 Jan 10;13:25. doi: 10.1038/s41467-021-27631-8 (PMC8748719; doi:10.1038/s41467-021-27631-8)
Supplement: Supplementary file 3 — Description of Additional Supplementary Files [file 41467_2021_27631_MOESM3_ESM.docx]

Description of Additional Supplementary Files

File Name: Supplementary Information.
Description: plots drawn using UpsetR package (R software version 3.3 (R project, worldwide release)) representing overlaps between drug classes in the whole dataset (Supplementary Figure 1), between drug substances in the antipsychotic group (Supplementary Figure 2) and salicylate group (Supplementary Figure 3), between subclasses in immunotherapy (Supplementary Figure 4) and cytotoxic (Supplementary Figure 5) and types of vaccines in the vaccine group (Supplementary Figure 6).

File Name: Supplementary dataset 1.

Description: Cases description, by drug substance and relevant subgroups analysis.

File Name: Supplementary dataset 2.

Description: Cases description, by drug substance.

File Name: Supplementary dataset 3.

Description: Pharmacovigilance causality assessment of drug substances not previously associated with myocarditis in their Food and Drugs Administration (FDA) label description.

File Name: Supplementary dataset 4.

Description: Cases descriptions, by drug class, with heatmap of associated adverse drug reactions (green to red, least to most associated).

File Name: Supplementary dataset 5.

Description: Categorization of MedDRA preferred terms regarding concurrent adverse events
