# Supplementary material for: Systematic analysis of drug-associated myocarditis reported in the World Health Organization pharmacovigilance database
Source: Nat Commun. 2022 Jan 10;13:25. doi: 10.1038/s41467-021-27631-8 (PMC8748719; doi:10.1038/s41467-021-27631-8)
Supplement: Supplementary file 9 — Reporting Summary [file 41467_2021_27631_MOESM9_ESM.pdf]

nature portfolio

Corresponding author(s): DBPR-NCOMMS-21-05826C

Last updated by author(s): Nov 18, 2021

Reporting Summary

Nature Portfolio wishes to improve the reproducibility of the work that we publish. This form provides structure for consistency and transparency in reporting. For further information on Nature Portfolio policies, see our Editorial Policies and the Editorial Policy Checklist.

Statistics

For all statistical analyses, confirm that the following items are present in the figure legend, table legend, main text, or Methods section.

n/a	Confirmed	
☐	☒	The exact sample size (n) for each experimental group/condition, given as a discrete number and unit of measurement
☐	☒	A statement on whether measurements were taken from distinct samples or whether the same sample was measured repeatedly
☐	☐	The statistical test(s) used AND whether they are one- or two-sided<br>*Only common tests should be described solely by name; describe more complex techniques in the Methods section.*
☐	☒	A description of all covariates tested
☐	☒	A description of any assumptions or corrections, such as tests of normality and adjustment for multiple comparisons
☐	☒	A full description of the statistical parameters including central tendency (e.g. means) or other basic estimates (e.g. regression coefficient) AND variation (e.g. standard deviation) or associated estimates of uncertainty (e.g. confidence intervals)
☐	☒	For null hypothesis testing, the test statistic (e.g. F, t, r) with confidence intervals, effect sizes, degrees of freedom and P value noted<br>*Give P values as exact values whenever suitable.*
☒	☐	For Bayesian analysis, information on the choice of priors and Markov chain Monte Carlo settings
☒	☐	For hierarchical and complex designs, identification of the appropriate level for tests and full reporting of outcomes
☒	☐	Estimates of effect sizes (e.g. Cohen's d, Pearson's r), indicating how they were calculated

Our web collection on statistics for biologists contains articles on many of the points above.

Software and code

Policy information about availability of computer code

Data collection	VigiLyze in VigiBase(r)
Data analysis	Disproportionality analyses were performed using VigiLyze, an integrated software with the VigiBase pharmacovigilance database. Data management and graphical representation was performed using Python version 3.0 (Python Software Foundation, Delaware, United States of America).

For manuscripts utilizing custom algorithms or software that are central to the research but not yet described in published literature, software must be made available to editors and reviewers. We strongly encourage code deposition in a community repository (e.g. GitHub). See the Nature Portfolio guidelines for submitting code & software for further information.

Data

Policy information about availability of data

All manuscripts must include a data availability statement. This statement should provide the following information, where applicable:

- Accession codes, unique identifiers, or web links for publicly available datasets
- A description of any restrictions on data availability
- For clinical datasets or third party data, please ensure that the statement adheres to our policy

Provide your data availability statement here.

Field-specific reporting

Please select the one below that is the best fit for your research. If you are not sure, read the appropriate sections before making your selection.

- [x] Life sciences
- [ ] Behavioural & social sciences
- [ ] Ecological, evolutionary & environmental sciences

For a reference copy of the document with all sections, see nature.com/documents/nr-reporting-summary-flat.pdf

Life sciences study design

All studies must disclose on these points even when the disclosure is negative.

Sample size	21 million reports. Statistical association observed in disproportionality analyses are based on the large sample size of the database (several millions). Hence, the sample size which comprises more than 21 millions reports is considered sufficient.
Data exclusions	None.
Replication	Replication as in the sense of performing the same analyses, may be applicable if a fellow researcher requests the exact same dataset (which is possible if asked upon the Uppsala Monitoring Centre in Sweden, with the proper date of extraction, mentionned in our manuscript, and the terms which were looked for, mentionned also in the manuscript) and performs the same analyses.
Randomization	Randomization was not applicable as this is an observational study.
Blinding	Blinding is not possible in a worldwide vigilance design study.

Reporting for specific materials, systems and methods

We require information from authors about some types of materials, experimental systems and methods used in many studies. Here, indicate whether each material, system or method listed is relevant to your study. If you are not sure if a list item applies to your research, read the appropriate section before selecting a response.

Materials & experimental systems

n/a	Involved in the study	
x		Antibodies
x		Eukaryotic cell lines
x		Palaeontology and archaeology
x		Animals and other organisms
x		Human research participants
	x	Clinical data
x		Dual use research of concern

Methods

n/a	Involved in the study	
x		ChIP-seq
x		Flow cytometry
x		MRI-based neuroimaging

Clinical data

Policy information about clinical studies

All manuscripts should comply with the ICMJEguidelines for publication of clinical research and a completedCONSORT checklist must be included with all submissions.

Clinical trial registration	NCT03855982
Study protocol	https://clinicaltrials.gov/ct2/show/NCT03855982
Data collection	This is a worldwide observational case-non-case cross-sectional study focusing on drug-induced myocarditis using the international pharmacovigilance database, VigiBase® (NCT03855982).13 VigiBase® is the WHO global individual case safety reports (ICSR) deduplicated database, managed by the Uppsala-Monitoring-Centre (Uppsala, Sweden)(accessible at www.vigiaccess.org). It contains over 21 million ICSR received from over 130 countries since 1967 with over 25,000 drugs and vaccines. ICSR originate from different sources, such as healthcare professionals, patients, and pharmaceutical companies, and are generally notified post-marketing. ICSR include administrative information (country, type of report and reporter), patient data (age, sex), date of onset of reaction(s) and nature of the outcome using the latest version of MedDRA (Medical Dictionary for Regulatory Activities) terms (currently v22.1).
Outcomes	We identified cases of myocarditis by searching in VigiBase® all ICSR flagged with the MedDRA preferred-term level « myocarditis » from inception to January, 12th, 2020; with a drug declared as "suspect" or "interacting" with myocarditis reaction. To do so, we used the VigiLyze software with the English version 22.1 of MedDRA (Uppsala Monitoring Centre, Sweden).<br>The main outcome was the number of myocarditis induced by drugs and report of cases of myocarditis associated with drugs.
